# The Cardiac Mineralocorticoid Receptor (MR): A Therapeutic Target Against Ventricular Arrhythmias

**DOI:** 10.3389/fendo.2021.694758

**Published:** 2021-06-28

**Authors:** Michel F. Rossier

**Affiliations:** ^1^ Service of Clinical Chemistry & Toxicology, Hospital of Valais, Sion, Switzerland; ^2^ Department of Medicine, Faculty of Medicine, University of Geneva, Geneva, Switzerland

**Keywords:** mineralocorticoid receptor, aldosterone, ventricular cardiomyocytes, arrhythmias, fetal gene re-expression, T-type calcium channels, steroid signaling, oxidative stress

## Abstract

Mineralocorticoid antagonists have been shown to be useful in the treatment of severe heart failure and may even save lives in this context. However, the reason for the beneficial action of these drugs, as well as the physiological role played by the cardiac mineralocorticoid receptor (MR), are still poorly understood. While the proinflammatory action of aldosterone on the heart and the resulting fibrosis partly explain the improvement due to the anti-mineralocorticoid therapy, the reduction in sudden death is probably related to a lower occurrence of ventricular arrhythmias. In this review, the author explains the physiological mechanism linking the positive chronotropic response induced by aldosterone observed *in vitro* with isolated ventricular cardiomyocytes and the increased risk of ventricular arrhythmias reported *in vivo* in hyperaldosteronism. He describes the molecular steps involved between MR activation and acceleration of spontaneous myocyte contractions, including expression of a specific micro RNA (miR204), down-regulation of a silencing transcription factor (NRSF), and re-expression of a fetal gene encoding a low threshold voltage-gated calcium channel (CaV3.2). Finally, he provides evidence suggesting aldosterone-independent and redox-sensitive mechanisms of MR activation in cardiac myocytes. Taken together, this information suggests that the use of anti-mineralocorticoid therapy could benefit the heart by preventing ventricular arrhythmias, not only in established hyperaldosteronism, but also in various pathological situations such as Cushing’s disease, oxidative stress, or even diabetes mellitus.

## Introduction

The mineralocorticoid receptor (MR), also called aldosterone receptor, is a nuclear receptor of the subfamily 3 (NR3C2), a group of proteins comprising the receptors for steroid hormones. This receptor principally works as a ligand-activated transcription factor, controlling the expression of several hundred different genes, but it also exerts non-genomic actions outside the cell nucleus. Paradoxically, MR displays a similar high affinity for mineralocorticoids (like aldosterone) and glucocorticoids (like cortisol), and its selectivity for aldosterone in some tissues, such as the distal nephron of the kidney, is achieved through a local preferential metabolism and inactivation of glucocorticoids by a specific enzyme, the 11β-hydroxysteroid dehydrogenase type 2 (11β-HSD2).

Classically, MR activation leads to the expression of proteins regulating ionic and water transport in several organs. Sodium reabsorption induced by MR, if inappropriate, results in increased blood pressure and high risk of hypokalemia. For this reason, synthetic MR antagonists, both steroidal (spironolactone, canrenone, eplerenone) and non-steroidal drugs (apararenone, esaxerenone, finerenone), have been developed for preventing adverse effects of aldosterone excess.

The publication of the RALES (for Randomized Aldactone Evaluation Study) in 1999 (1) has been a true revelation for cardiologists and endocrinologists. The authors demonstrated for the first time the clear beneficial effect of low dose of spironolactone, a well-known antagonist of MR, in the treatment of severe heart failure. Indeed, the addition of the antagonist on top of a classical treatment for this pathology, including ACE inhibitors, loop diuretics, and digoxin, reduced the mortality by 30% at 3 years. The benefice of spironolactone was so obvious that the study was discontinued early in order to propose this treatment to all patients.

The involvement of MR in the pathophysiology of heart dysfunction was then further confirmed in another study (called EPHESUS for Eplerenone Post–Acute Myocardial Infarction Heart Failure Efficacy and Survival Study) employing eplerenone instead of spironolactone (2). This selective aldosterone blocker also improved drastically the outcome of patients with acute myocardial infarction complicated by left ventricular dysfunction and heart failure. In particular, a significant reduction in the rate of sudden death from cardiac causes was observed in the eplerenone group.

Importantly, the beneficial action of MR antagonism on the cardiac function was independent of blood pressure improvement or serum potassium normalization, suggesting that these drugs were acting on mechanisms distinct from the classical effect of mineralocorticoids leading to sodium reabsorption (3).

Besides the promotion of inflammation and fibrosis induced by aldosterone in the heart and vasculature (4–6), explaining in part the reduction of morbidity in heart failure by MR antagonists, aldosterone has been also shown to be responsible for electrical remodeling and cardiac dysfunction (3, 7). Indeed, several studies reported a significant association between hyperaldosteronism resulting from adrenal adenoma and the risk of cardiovascular (CV) events, in particular sudden death due to ventricular fibrillation (8–11). Hyperaldosteronism therefore appeared as a newly described cause of sudden cardiac death, which should be considered in patients with malignant arrhythmias and hypokalemia.

**Figure 1:**
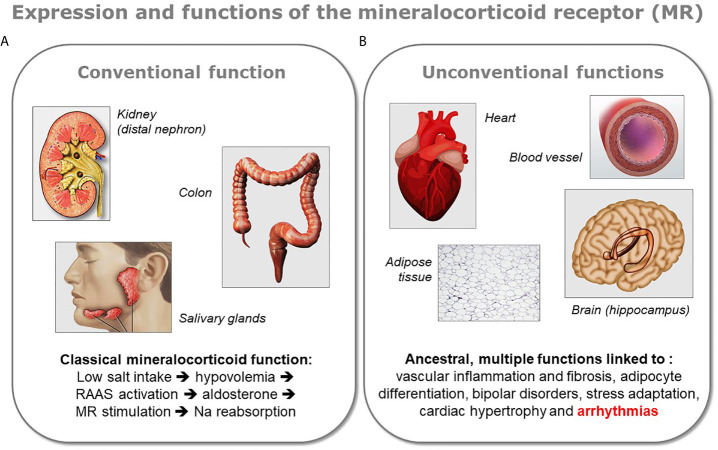
Conventional and unconventional MR functions. The various functions of the mineralocorticoid receptor (MR) can be divided in two groups depending on the organ expressing the protein. **(A)** The receptor exerts its classical mineralocorticoid function in tight epithelia present in the distal nephron of the kidney, in colon, or in salivary gland, where it facilitates sodium reabsorption upon activation of the renin-angiotensin-aldosterone system (RAAS). **(B)** MR is also expressed in other organs like the brain (mostly in the hippocampus), the heart, the blood vessels, or the adipose tissue. In the latter organs the role of MR is multiple and less well characterized, however a dysregulation of the receptor has been shown to be linked to several pathologies. Because the presence of MR in these organs appeared in the evolution before the classical mineralocorticoid function associated to aldosterone, these unconventional functions of MR are considered as ancestral.

The demonstration of the expression of MR in the heart, as well as in other organs not involved in the control of sodium balance, led recently to a revision of our understanding of MR physiology (**Figure 1**). Indeed, MR is classically recognized for its crucial role in the evolution because of the need for salt preservation by living beings when they began the colonization of the emerged lands. This function is closely related to the development of various key organs such as the kidney, colon, and salivary glands in these organisms. However, besides this function, fundamental for the evolution of species on the earth, there exist other roles for this receptor, probably ancestral and certainly more obscure. These “unconventional” functions of MR are exerted in various organs like brain, heart, blood vessels, or adipose tissue.

Interestingly, MR appeared in the evolution approximately 460 M years ago, as the result of a duplication of a common ancestral receptor shared with the glucocorticoid receptor (GR), and MR is found in several fish branches, demonstrating its presence a long time before the appearance of aldosterone (12). Indeed, when aldosterone appeared later in the evolution, with the first tetrapods, MR was already expressed in the brain and heart of fishes. In spite of the absence of aldosterone in fishes, their MR can respond to the addition of the exogenous hormone or to steroid analogs. The nature of the MR endogenous ligand in older species like fishes, as well as the function of this receptor in the heart, are still a matter of debate (13).

The aims of this mini-review are discussing a recently developed molecular model for linking the activation of the cardiac MR to the electrical remodeling of the cardiomyocytes responsible for the increased risk of ventricular arrhythmias, and discussing the nature of the ligand(s) and mechanisms leading to receptor stimulation.

## The Chronotropic Action of Aldosterone on Isolated Rat Cardiomyocytes Is Due to Cell Electrical Remodeling

When isolated neonate rat ventricular cardiomyocytes, maintained in primary culture, are exposed to exogenous aldosterone, one can observe a strong and robust acceleration of their spontaneous beatings (14, 15). This chronotropic action of aldosterone on isolated cardiomyocytes *in vitro* is certainly relevant for understanding the pathophysiology of the aldosterone-induced ventricular arrhythmias *in vivo*. Indeed, spontaneous depolarization and contraction of a group of ventricular muscle cells can be a source of local arrhythmias in the heart, particularly if occurring before the propagation of the signal induced by the cardiac pacemaker.

In fact, upon cardiac development, ventricular cells lose their ability to contract spontaneously as the pacemaker takes control of the sinus rhythm. However, under some circumstances like in case of chronic heart failure, ventricular cells can reactivate a program of fetal gene expression and reacquire the faculty of contracting spontaneously (16, 17). In this situation, any chronotropic agent acting on these cells would increase de risk of ventricular arrhythmias.

The knowledge of the molecular mechanisms responsible *in vitro* for the acceleration of isolated cardiomyocyte spontaneous contractions upon aldosterone stimulation therefore appears mandatory for understanding the relationship observed clinically between an excess of this hormone and the increased incidence of sudden death.

The chronotropic action of aldosterone is observable only after 18 h incubation, suggesting a complex genomic effect of the hormone on the cardiomyocytes (15). Indeed, actinomycin D, a blocker of gene transcription, completely abolishes aldosterone action, which is also prevented in the presence of spironolactone, demonstrating the involvement of MR in this response (15, 18). A putative implication of GR is also suggested by two additional observations. A partial inhibition of the response to aldosterone is obtained with the GR-unspecific antagonist, RU-486, and the chronotropic effect of aldosterone still increases at supra-physiologic concentrations of the hormone (above 1 micromolar) where MR is expected to be fully saturated and the low affinity GR starts to be activated by this steroid (14, 18).

Aldosterone induces, after 24 h stimulation, a significant increase of the amplitude of the calcium currents in cardiomyocytes, with an effect more pronounced at negative voltages, suggesting that the additional current is mostly carried by low threshold T-type calcium channels (14, 19). Accordingly, within the same period, aldosterone increases the level of mRNA coding for the alpha1H protein, which forms the core of the CaV3.2 channel, one of the two T channel isoforms expressed in cardiomyocytes. Moreover, preventing alpha1H expression with specific small interfering RNA (siRNA) abolishes the chronotropic response to aldosterone demonstrating the crucial role played by this channel (19).

**Figure 2:**
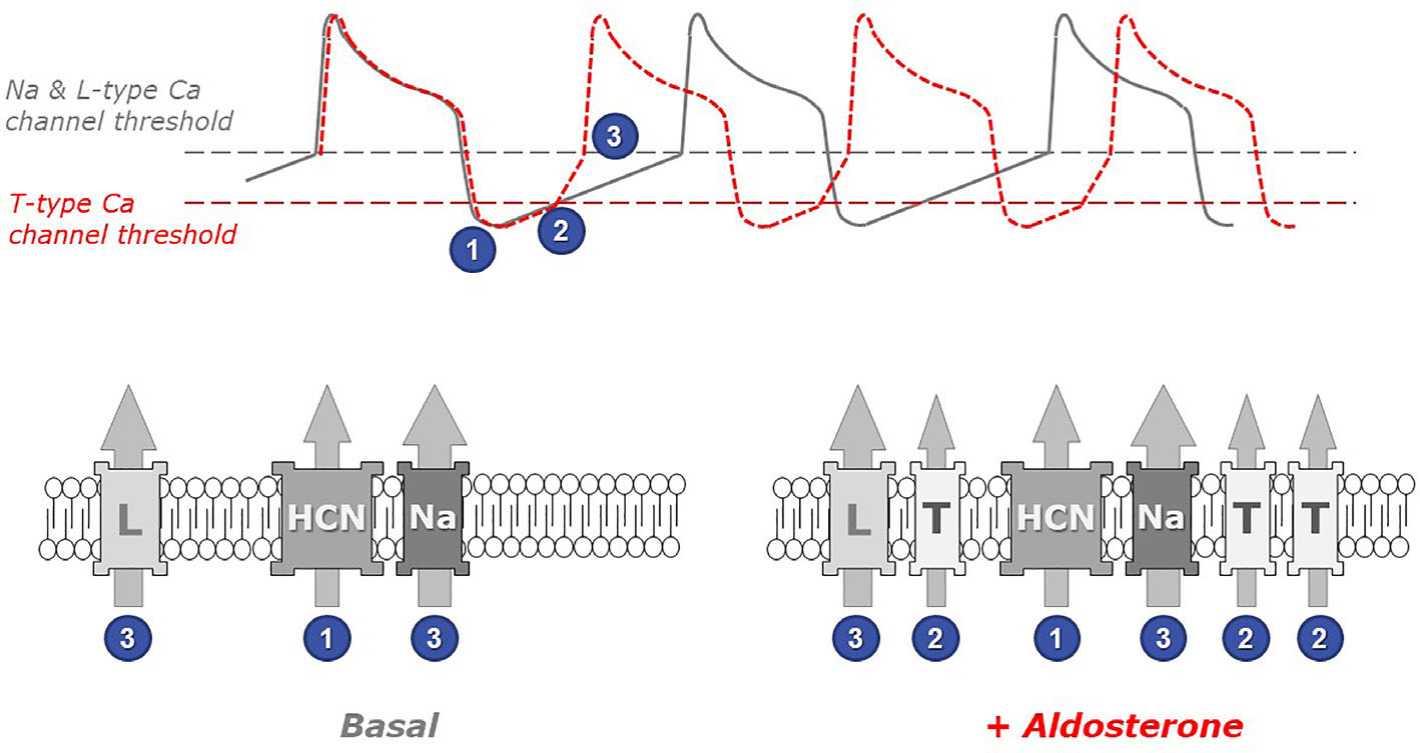
The role of low threshold T-type calcium channels in the chronotropic response to aldosterone. Spontaneous cardiomyocyte beatings are closely linked to action potentials (APs) occurring when the membrane voltage reaches the threshold for Na and L-type Ca channel activation (upper panel, gray line). Under resting (basal) conditions, the frequency of APs depends on the duration of the diastolic depolarization phase (following the transient hyperpolarization occurring at the end of each AP), which is principally controlled by the activity of pacemaker channels such as the HCN channels, which open at very negative voltages (step 1 in figure). Upon myocyte stimulation with aldosterone (red line), the presence of low threshold T-type Ca channels in myocyte membranes accelerates the occurrence of APs. Indeed, these channels open early during the depolarization phase (step 2) and therefore contribute to membrane depolarization. Thus, the membrane potential reaches earlier the threshold for triggering the next action potential (step 3). The gray dotted line in the upper panel indicates the voltage at which Na and L-type Ca channels activate, while the red dotted line indicates the (lower) threshold of T-type Ca channel activation. The identity of relevant ionic channels present in cardiomyocyte membranes under basal conditions (left) and in aldosterone-stimulated myocytes (right) is shown in the bottom panels: HCN, hyperpolarization-activated cyclic nucleotide-gated cation non-selective (pacemaker) channel; Na, voltage-activated sodium channel; L and T, high-threshold L-type and, respectively, low-threshold T-type, voltage-activated calcium channels. Numbers indicate at which step each channel activates.

The functional link between the overexpression of this low voltage-activated calcium channel and the acceleration of the spontaneous cell beatings is straightforward. Indeed, the beating frequency is dictated and inversely proportional to the duration of the diastolic period between two consecutive action potentials (**Figure 2**).

Cardiomyocyte contraction is induced by calcium entry and calcium release from intracellular stores occurring upon each action potential. Immediately after an action potential, the cell is temporarily hyperpolarized and must progressively return to more depolarized potentials in order to reach the voltage threshold that will trigger the next action potential. The beating frequency is therefore controlled by the rate of membrane depolarization occurring between two action potentials. This depolarization mechanism, in resting cells, is mostly due to the activity of specific pacemaker channels, called HCN and carrying positive charges into the cells. Because of their low threshold of activation, T-type calcium channels (expressed upon aldosterone exposure) open early during this diastolic phase and therefore add their contribution to the cell depolarization. In consequence, the next action potential (and the corresponding contraction) happens earlier.

## Signaling Pathway Between MR Activation and T Channel Expression

While the link between the overexpression of low threshold T-type calcium channels and the acceleration of myocyte beatings is easy to understand, in contrast, the mechanism by which aldosterone controls the expression of alpha1H is much less obvious. *In vivo* transcriptomic studies revealed that overexpression of MR in mouse cardiomyocytes leads to the differential regulation of several hundreds of genes and 43 of those are modulated by aldosterone (20). However, because animals were treated 7 days with aldosterone, responding genes were not necessarily primary targets of MR, but rather genes regulated subsequently to the chronic activation of the receptor.

Moreover, the promoter of the gene CACNA1H coding for the CaV3.2 T-type channel does not contain any mineralocorticoid response element (MRE), a consensus nucleotide sequence functioning as anchoring domain for MR (21). This lack of MRE suggests that the control of channel expression by aldosterone is probably indirect and more complex. In this context, aldosterone has been shown to induce a significant expression of a specific micro RNA, miR-204, already markedly elevated after 3 h incubation with aldosterone and maintained high after 24 h incubation (21). Spironolactone completely abolishes the response to aldosterone demonstrating that miR-204 expression depends on MR activation.

Moreover, miR-204 overexpression by transfection in cardiomyocytes mimics aldosterone effect on the beating frequency, as well as on alpha1H channel expression and T-type calcium current induction. In contrast, transfecting cells with a miR-204 antagomir prevents aldosterone action on these same parameters (21).

**Figure 3:**
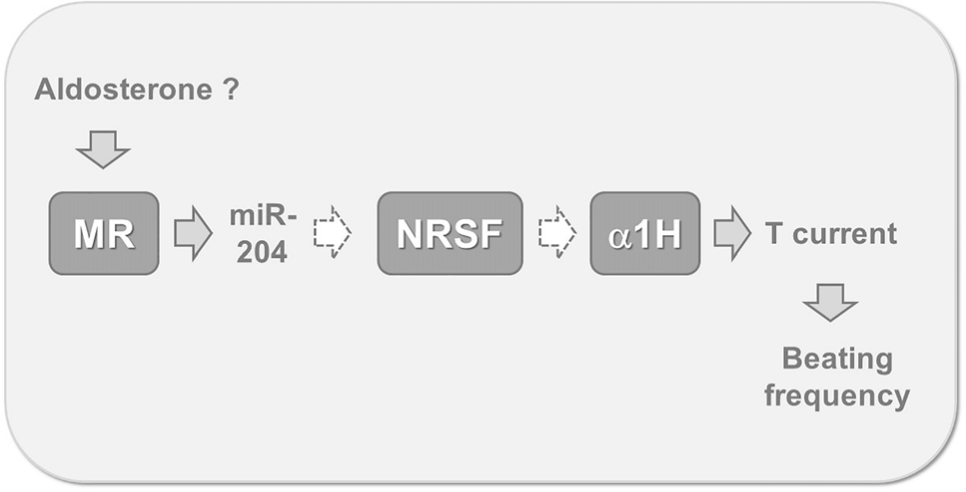
Putative mechanism linking MR activation to increased myocyte beating frequency. Recent findings suggest that cardiomyocyte MR stimulation, by aldosterone or other ligands, leads to the expression of miR-204. This micro RNA reduces the translation of mRNA coding for NRSF, a specific repressor maintaining low levels of alpha 1 H expression. This mechanism is globally responsible for T channel overexpression, for an increased inward T-type calcium current during the diastolic phase and therefore for the acceleration of spontaneous beatings. Gray arrows indicate stimulatory, while white dotted arrows indicate inhibitory steps. MR, cardiac mineralocorticoid receptor; miR-204, micro RNA 204; NRSF, neuron-restrictive silencing factor; α1H, the alpha 1 subunit of the CaV3.2 T-type calcium channel; T current, calcium current supported by low threshold T-type calcium channels.

It can therefore be proposed that miR-204 acts downstream of MR for controlling T channel expression (**Figure 3**). However, because micro RNAs are supposed to exert a *negative* action on protein expression, one must invoke a second intermediate within the signaling pathway, controlled by miR-204 and exerting itself a *negative* action on alpha1H expression. The protein NRSF (which is for Neuron-Restrictive Silencing Factor) has been previously shown to be a chronic repressor of T channels in cardiomyocytes (22). This protein appears constitutively expressed in cardiac cells and abolished by both aldosterone and miR-204, while its messenger RNA remains unaffected by these treatments, a feature typical of the action exerted by micro RNAs (21). A complete putative signaling pathway linking aldosterone down to cardiomyocyte beating acceleration is proposed on **Figure 3**. Whether the miR-204 promoter, located on TRPM3 (Transient Pore Receptor Member 3) gene (23), possesses specific steroid hormone-response elements, which MR binds to for regulating gene expression, remains to be determined.

## Mechanisms of Aldosterone-Independent Activation of Cardiac MR

The functional characterization of MR revealed that this receptor has a very high affinity for both aldosterone and cortisol (or corticosterone in rodents), with Kd values for ligand binding around the nanomolar values (24). It has been therefore proposed that MR has, in reality, two physiological hormonal agonists: not only aldosterone, but also cortisol (25). Under these conditions, one can predict that MR should constantly be occupied by glucocorticoids, which circulate at physiological concentrations 100 to 1,000 times higher than those of mineralocorticoids, and therefore that glucocorticoids prevent aldosterone-induced receptor modulation. This apparent paradox has been solved in kidney by the discovery of the role of the 11β−HSD2 enzyme. This protein converts cortisol into cortisone or, in rodents, corticosterone into its 11-keto form, metabolites displaying very low affinity for MR. This MR protective mechanism is efficient in late distal nephron where aldosterone can thus exert its mineralocorticoid action in spite of the presence of large amounts of glucocorticoids (26, 27). However, this protection is apparently lacking in cardiac myocytes, where very little 11β−HSD2 activity has been measured, meaning that the cardiac MR should be primarily occupied by glucocorticoids (28–30).

Interestingly, addition of corticosterone *in vitro* can mimic the chronotropic action of aldosterone on isolated rat cardiomyocytes only when the cells are previously exposed to an oxidative stress (15). Indeed, in the absence of oxidant no response to the glucocorticoid is observed and no effect either is obtained with the oxidant alone. These experiments have been performed systematically in the presence of RU-486, in order to block GR activity in myocytes, and the involvement of MR in this oxidation-dependent chronotropic action of corticosterone has been confirmed by its complete inhibition by spironolactone (15).

This requirement of combining both glucocorticoid binding and cell oxidation to activate this receptor suggests that MR could play the role of a redox sensor in the heart. Indeed, the high affinity of the receptor for glucocorticoids is not adapted for sensing variations of the concentrations of this hormone in the blood because the receptor is supposed to be always saturated by this ligand (28). However, in contrast to aldosterone, the presence of corticosterone on MR is not sufficient for activating the receptor and inducing the program of gene expression leading to membrane electrical remodeling. While the presence of the glucocorticoid ligand is apparently here only for the “priming” of the receptor, the real trigger for activating the program is in fact the oxidative stress. This mechanism possibly reflects an ancestral function of MR in the evolution, present a long time ago, before the appearance of aldosterone, which appeared later as a first full agonist of this receptor.

Glucocorticoid hormones are often considered clinically as cardioprotective because of their ability to antagonize aldosterone action (31–33). However, deleterious effects of these hormones on the cardiac function have been also reported. Indeed, high serum cortisol is an independent predictor of mortality in patients with heart failure (34). Moreover, MR antagonism is beneficial for preventing cardiac hypertrophy and failure also in hypertensive rat with low aldosterone (35), suggesting that in this particular situation MR is not activated by aldosterone itself but through glucocorticoid binding. The possibility that a change of the cellular redox state transforms glucocorticoids from antagonists to agonists for MR has been previously proposed by Funder (28). This hypothesis is particularly relevant given the severe damage due to reactive oxygen species occurring after myocardial infarction, upon cardiac ischemia reperfusion injury (36, 37) and the therapeutic role proposed for antioxidants (38). The manifestations of reperfusion injury include arrhythmias, myocardial stunning, and micro-vascular dysfunction, in addition to significant cardiomyocyte death (39). Moreover, oxidative stress has been reported to be responsible for the left ventricular remodeling resulting from myocardial infarction (40). Pilot studies have shown a strong cardioprotective effect of MR antagonists, when added before reperfusion, both in experimental animal models (41) and in humans (42).

Diabetes mellitus, a pathology associated with significant cardiovascular morbidities, increases the risk of cardiac arrhythmias (43). Indeed, ventricular arrhythmias are thought to underlie sudden cardiac death in type 2 diabetic patients. In this context, it has been recently shown that glucose exerts *in vitro* a concentration-dependent chronotropic action on isolated rat ventricular myocytes (44). Remarkably, the response to glucose appears only several hours after cell exposure and is completely abolished by spironolactone or eplerenone, demonstrating the involvement of MR in this effect. However, in contrast to the action of aldosterone, the chronotropic response to glucose is insensitive to actinomycin D and does not imply alpha1H expression. These discrepancies speak in favor of the existence of different mechanisms of action for both agents.

A similar chronotropic effect of high glucose levels on isolated cardiomyocytes has been reported by several authors and various mechanisms have been proposed (45, 46). Interestingly, high glucose is recognized as an inducer of oxidative stress in different models of cardiomyocytes from various species (47, 48). Oxidative stress is linked to ROS production, DNA damage, and increased beating frequency, all being adverse effects considered as potential cardiotoxic responses to hyperglycemia. Given the putative role played by the cardiac MR as stress sensor in the response of cardiomyocytes to oxidation, it would be relevant to test the protective effect MR antagonists in these models.

## Discussion

The mineralocorticoid receptor is not only expressed in epithelial tissues where it is known to exert its “conventional” function on sodium reabsorption and potassium excretion, but also in other organs like the brain or the cardiovascular system. While the exact role of MR in the latter remains to be elucidated, overstimulation of this receptor can lead to deleterious conditions, such as tissue inflammation, fibrosis, and apoptosis, as well as, in the case of the heart, to electrical remodeling and hypertrophy. In this context, hyperaldosteronism has been linked clinically to an increased risk of ventricular arrhythmias and sudden death, independently of the blood pressure or serum potassium concentration, plenty justifying the utilization of MR antagonists like spironolactone and eplerenone in the treatment of cardiac failure.

The molecular link between MR activation and cell electrical remodeling has been elucidated during the last decade. These studies revealed the role of a specific micro RNA, negatively modulating the levels of a transcription silencing factor and therefore enabling the re-expression of fetal genes, including a low threshold calcium channel (**Figure 3**). This mechanism provides a strong basis for explaining the chronotropic action of aldosterone, determined *in vitro* on isolated cardiomyocyte, and possibly linked to the increased rate of arrhythmias observed *in vivo*.

Paradoxically, in the heart, *glucocorticoids* are expected to constitutively bind MR, preventing activation of the receptor by physiological concentrations of aldosterone. The binding of cortisol (or corticosterone in rodents) however, is insufficient for activating the program initiated by the receptor that requires triggering elicited by a change of the redox state in the cell. This complex mechanism of receptor activation possibly reflects ancestral function of MR, present in the evolution of species a long time before the appearance of aldosterone. The activation of this receptor by glucose, probably indirectly through modulation of the cell oxidation, highlights the relevance of this functional genomic response.

Extrapolating data obtained *in vitro* with an animal model into clinical practice always needs much caution. Nevertheless, the marked beneficial action of MR antagonism demonstrated in the treatment of cardiac failure, often in the absence of elevated aldosterone or sodium retention, strongly suggests that a similar therapeutic approach would be worth of investigation in the context of different pathophysiological situations, including cardiomyopathies associated with diabetes mellitus, Cushing syndrome, or reperfusion after ischemia. This assumption is somehow supported by the recent use of novel, selective non-steroidal MR antagonists, like finerenone (49, 50), in the management of diabetic nephropathy and associated cardiovascular risk.

## Author Contributions

MR is responsible for the complete elaboration, writing, and correction of the present manuscript.

## Conflict of Interest

The author declares that the research was conducted in the absence of any commercial or financial relationships that could be construed as a potential conflict of interest.

## Abbreviations

MR, mineralocorticoid receptor; GR, glucocorticoid receptor; ACE, angiotensin I converting enzyme; siRNA, small interfering RNA; HCN, hyperpolarization-activated cyclic nucleotide-gated cation non-selective (pacemaker) channel; AP, action potential; CV, cardiovascular; miR-204, micro RNA 204; NRSF, neuron-restrictive silencing factor; MRE, mineralocorticoid response element; α1H, alpha1 subunit of the CaV3.2 T-type calcium channel; T current, calcium current supported by low threshold T-type calcium channels; 11β−HSD2, 11-beta-hydroxysteroid dehydrogenase of type-2; ROS, reactive oxygen species.
